# PROTOCOL: Interventions to promote technology adoption in firms: a systematic review

**DOI:** 10.1002/CL2.206

**Published:** 2018-07-30

**Authors:** Eric Verhoogen, David Alfaro‐Serrano, Tanay Balantrapu, Ana Goicoechea

## Background

### The problem, condition or issue

The adoption of improved technologies is associated with better economic performance and development in general. However, there is evidence that the market processes alone are not enough to induce firms to adopt the best technologies available.

Governments and development agencies have incorporated the promotion of firms’ competitiveness into their priorities (World Bank, 2017; IADB, 2016) and recognized that the adoption of modern technologies is one of its drivers. Two reasons lie behind this interest in competitiveness in general and technological upgrading in particular: first, the expectation that technological upgrading by firms will deliver benefits like higher productivity, more jobs, better wages and better working conditions at the micro level, and higher growth at the aggregate level; second, the idea that the slowness, or even absence, of the process of technology adoption is due in part to market failures that call for public intervention, like externalities, imperfect information and coordination problems.

History provides examples of major increases in wellbeing associated with the widespread adoption of improved productive methods. In manufacturing, we point to the so‐called three industrial revolutions (OECD, 2017; Cirera et al, 2017): the introduction of steam‐powered machines, the adoption of electricity‐powered production methods, and the use of information technologies to automate manufacturing. In agriculture, the green revolution had a comparable impact. The key aspect in these revolutionary transformations was not the mere invention of new technologies, but their widespread adoption by the productive units.

Despite its desirable effects, there is evidence that market processes alone are not enough to induce firms to adopt the best technologies among those available, and when it happens, the process can be quite slow (Geroski, 2000; Rosenberg, 1972; Griliches, 1957). The lack of technology adoption in cases in which the potential gains are clear have been observed in specific industries like textiles (Bloom et al, 2013) and soccer ball production (Atkin et al, 2017). Foster, Haltiwanger and Syverson (2008) show that there is large heterogeneity in productivity even within narrowly defined industries, which may be the consequence of the lack of technology adoption by the firms in the left tail of the distribution.

To help inform governments’ actions, this systematic review will aggregate the existing evidence on interventions to promote technology adoption and its implication for firm outcomes.

### The intervention

This review will focus on interventions that induce adoption of a new technology by a firm. Following Foster and Rosenzweig (2010), we define *technology* as “the relationship between inputs and outputs”, and *technology adoption* as “the use of new mappings between input and outputs and the corresponding allocations of inputs that exploit the new mappings”. This definition of technology adoption is broad, including the overall production plan that firms implement (the recipe they use) as well as changes in firm's practices. Evenson and Westphal (1995) differentiate between these two concepts, giving the label technology to the former and technique to the latter. We subsume both under the term *technology*.

The word intervention is considered broadly, including public interventions, interventions carried out by private institutions (like NGOs), experimental variations deliberately induced by academic researchers trying to understand technology adoption, and quasi‐experimental changes (including natural experiments) exploited in studies about technological change. The interventions of interest are defined by their effect: to induce technology adoption by firms. Even though this review is not focused on a particular type of policy, it is possible to provide a broad classification of the interventions we expect to find. As pointed by Cirera et al (2017), technology adoption is one type of innovation. Consequently, a taxonomy of innovation policies is useful to illustrate the variety of interventions we might find, even though it is possible that some of the interventions we might include are not actually implemented by governments. Here we follow one of the classifications presented by Cirera and Maloney (2017):

1. Indirect financial support. These are interventions that help businesses to pay for the cost of the adoption projects without directly providing the resources. Loan guarantees and fiscal incentives are examples of this type of intervention. Loan guarantees help overcome the problem of asymmetric information by reducing the risk perceived by the financier. Fiscal incentives might help overcome the obstacle derived from lack of appropriability.
2. Direct financial support. These are interventions that directly provide funding for technological adoption. An example of this type of intervention is the credit instrument for technological upgrading provided by the Argentinean FONTAR program (Castillo et al, 2014).
3. Other direct support. These are non‐pecuniary interventions. Within this category, we find policies implemented by governments like technology extension services and experimental interventions like the direct provision of management consultancy services implemented by Bloom et al (2013) and the awareness intervention implemented by Beaman et al (2014).
4. Regulation and standards. These are rules and characteristics of the environment that affect agents’ incentives. For example, the level of competition or the characteristics of contracts governing the relationship between employers and employees.

It is important to note that the focus of this review will be the effect of the interventions on technology adoption by *existing* firms. In this sense, the interventions considered are different from interventions to promote entrepreneurship. We expect that most of the studies to be included will be about the adoption of technologies that are new to the firm, but not to the world.

### How the intervention might work

**Figure 1:**
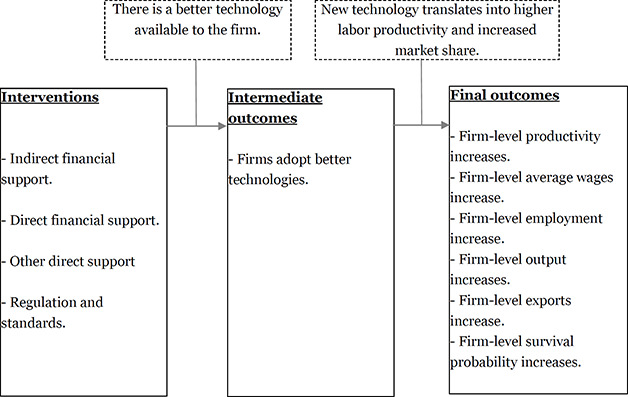
Theory of change

Figure 1 presents the theory of change of the interventions of interest. The solid boxes represent the intervention and outcomes and the dashed boxes represent the assumptions. As stated before, this review will not be devoted to a single type of intervention. Instead, it will include different interventions inducing technology adoption in firms. Despite their heterogeneity, it is useful to provide a general theory of change describing the way in which the interventions, intermediate outcomes and final outcomes relate.

In the previous section, we mentioned that, broadly speaking, these interventions can take the form of direct or indirect financial support, non‐pecuniary support, or regulation. These might induce the adoption of better technologies in the directly affected firms, provided that that there is a better technology available (after considering adoption costs) and that, after providing the treatment, firms are able to notice that advantage.

The adoption of a better technology by treated firms may lead to an increase in total output, output per unit of input, unit cost, firm‐level wages, employment, total factor productivity, firm survival, and/or exports. Productivity, understood as the inverse of cost, is expected to increase because the reduction in cost is what makes the new technology attractive to the firm. Output is expected to increase if a firm can increase its market share based on the improvement in cost. As consequence of the expansion in output, firm‐level employment is also expected to increase. Wages (although not necessarily wages *relative* to other input prices^1^) might also go up after adoption due to the rise in labor marginal productivity.

### Why it is important to do the review

Systematic reviews about technology adoption are mostly focused on agricultural firms (Obayelu et al, 2017; Waddington et al, 2014; Silva et al, 2015). There has been little effort to do the same for other economic sectors, despite the recent publication of papers on technology adoption in manufacturing (Bloom et al, 2013; Atkin et al, 2017). Consequently, existing summaries of evidence for nonagricultural firms are almost all nonsystematic literature reviews. Our review will address this knowledge gap by summarizing the existing evidence on technology adoption. For evidence related to agriculture, we will work in collaboration with the authors of the upcoming World Bank report “Understanding Productivity Growth in Agriculture.”

A review close to ours is the one by Piza et al. (2016). They systematically review the evidence about the impact of businesses‐support services for small and medium enterprises (SME) in low and middle‐income countries. Our review will complement their findings by broadening the types of countries and firms considered, and narrowing the type of intervention and outcomes to study. Including a broader set of firms is important because, even though SME are particularly relevant for outcomes like employment, larger firms are key for other important outcomes like exports. Considering also the experience of developed countries is warranted because active policies to promote technology adoption are widespread around the globe, not only in the developing world. The experience of these countries provides a knowledge collection relevant for policy making everywhere. Finally, our review's focus on technology adoption, leaving aside interventions aiming to affect other outcomes, will allow us to provide insight on the effectiveness of this particular type of policy. The rationale for public intervention in this area includes the existence of knowledge spillovers and self‐discovery (Hausmann and Rodrik, 2003), which are not present in the justification of other pro‐business interventions, like formalization and access to working capital.

Below we comment on the existing nonsystematic reviews on technology adoption by nonagricultural firms.

Herbert‐Copley (1990) reviews case studies of technical change in manufacturing firms in the 1980s in Latin America. In particular, the study assesses the role of the nature of technology, market structure, government policy, firm characteristics and the location of the international technological frontier on the level of technology adoption. Even though it is interesting, the study is outdated and its geographic coverage is limited.

Keller (2004) reviews technology diffusion, but only across countries. This review leaves out cases that will be relevant for our review, like technology diffusion from advanced firms to backward businesses within a country. It also obviously cannot speak to more recent evidence. For example, one of the author's conclusions is that there is no evidence of exports being a driver of technology improvement. However, there is recent evidence pointing in that direction (Bustos 2011; Atkin et al, 2017b).

Coming from the information systems literature, Oliveira and Martins (2011) compile studies on the adoption of information technology at firm level. The authors do an interesting job classifying different papers according to the model they based their analysis on. However, their review does not attempt to compare their results or to try to extract general lessons from them.

## Objectives

The objective of this review is to answer the following research questions:

1. To what extent do the interventions affect technology adoption in firms?
2. Is this effect heterogeneous across sectors, firm size, countries or owner's gender?
3. To what extent does technology adoption affect total output, output per unit of input, unit cost, firm‐level wages, employment, total‐factor productivity, exports, and survival?
4. Are these effects heterogeneous across sectors, firm size, countries, workers’ skill level, or workers’ gender?

Questions 1 and 2 refer to the immediate impact of interventions to promote technology adoption. Questions 3 and 4 explore the subsequent impact of technology adoption on other economic outcomes. Answering questions 2 and 4 will bring attention to the heterogeneity of the main effects and to potential unintended impacts. For example, it is possible that the effect of technology adoption on wages is negative in some sector or type of firm even though its overall average effect is positive.

Note that all the review questions take the productive unit as the unit of analysis. The reference to wages and employment in question 3 refers to firm‐level average and total level, respectively. Similarly, the reference to heterogeneity across workers’ skill level and gender in question 4, means that we will summarize information about firm‐level impacts on average wages and employment levels disaggregated by skill level and gender. Considering that some industrial surveys collect this type of firm‐level disaggregated information, it is possible that some of the papers included in the review also report effects disaggregated in these dimensions.

## Methodology

### Criteria for inclusion and exclusion of studies in this review

#### Types of participants

To be included, papers must study interventions affecting firms and must have productive units as their unit of analysis. This focus leaves out studies of technology adoption at country or region level, which is justified by the fact that the firm is the decision‐making unit regarding the adoption of a new productive method. Additionally, aggregate estimates would include not only the effects of the interventions on the firms, but also their effect on the composition of firms, which is outside the scope of this review.

This review will impose no restriction regarding the level of development of the country in which the intervention takes place. Developed countries have been active in promoting technological improvement for a long time. Now that this type of policy has spread around the world, their accumulated experience provides a knowledge stock valuable for policy‐making in other countries.

#### Interventions

The review will include studies about interventions that induce technology adoption by firms. To be included, studies should explicitly indicate this characteristic of the intervention. As stated in section 1.2, we define *technology* as “the relationship between inputs and outputs”, and *technology adoption* as “the use of new mappings between input and outputs and the corresponding allocations of inputs that exploit the new mappings”.

#### Outcomes

Following the theory of change presented in section 1.3, the outcome variables that will be considered in this review are classified in two groups: intermediate outcomes and final outcomes. Papers analyzing effects on final outcomes will be included only if they also present estimates of the effects on intermediate outcomes (technology adoption)^2^.

The intermediate outcomes are variables that reveal whether the firm adopted a new technology or not. These can take the form of a dichotomous variable that, for example, takes value one when a technology is adopted and zero otherwise; or a variable counting the number of new practices adopted.

The final outcomes are variables that are affected by technology adoption. These are variables in the second step of the causal chain. In particular, we will consider measures of output per unit of input, unit cost, wages, employment, output, total factor productivity, firm survival, and/or exports. Regarding measurement, we will require that i) output and output per worker are measured in physical or monetary units; ii) wages are measured in monetary units; iii) employment is measured in hours or days or number of employees; iv) exports are measured with an indicator variable that takes value one if the firms exported or by the monetary value of the sales to foreign customers; and iv) firm survival is measured by an indicator variable that takes value one if the firm is still operating.^3^ Total factor productivity will be treated as a dimensionless variable.

#### Comparisons

We will generally require that the comparison group is a set of productive units that received no treatment. Two sorts of exceptions to this rule will be considered acceptable. One is when the researchers provide some kind of compensation to the comparison group in order to replicate the non‐intervention situation or to isolate some aspect of the treatment. The other is when different groups receive exogenously different “doses”, or amounts, of treatment, but no group receives zero treatment. In these case, it is still possible to estimate causal effects cleanly, and hence they will be considered in the review.

#### Study design

The review will include studies that take the firm as the unit of analysis and that explicitly try to address the so called *fundamental problem of causal inference*.

The fundamental problem of causal inference is trying to disentangle the effect of the intervention of interest on a given outcome from the effect of other variables that also affect the outcome and correlate with the intervention. If these other variables are present, their influence on the outcome will be confounded with the influence of the intervention and the analyst could end up attributing to the treatment what is really the result of the omitted variable.

The gold standard to address the fundamental problem of causal inference is a randomized control trial. However, implementing one is sometimes impossible. Requiring a randomized experiment would be too strict and could lead to the exclusion of valuable research products. Consequently, in addition to experimental studies, we will also include papers using quasi‐experimental methods (e.g. instrumental variables and regression discontinuity designs) and non‐experimental methods (e.g. difference‐in‐difference, propensity score matching and synthetic control). For the studies using experimental and quasi‐experimental methods, we will only require that data from the moment of assignment and at least one follow up are presented. The data at the time of assignment helps to show that the identifying variation is random or quasi‐random. The follow up data is the one used to estimate the effects. For the studies using non‐experimental methods, data from at least one pre‐treatment point in time will be required. Regarding the level of assignment of the treatment, we will require that it is assigned at the level of the productive unit.

### Search strategy

The first step to find the studies to be included in the systematic review is identifying a set of *candidate papers*. This set should include both published and unpublished studies. To look for candidate papers, we will implement first an electronic search and in a subsequent step a manual search.

#### Electronic search

The sources of information to consider for the electronic searches will include bibliographic databases, web sites of relevant organizations and specialized journals.

The following bibliographic databases will be included in the search:

1. Academic Search Complete (through EBSCO Host).
2. Business Source Complete (through EBSCO Host).
3. EconLit (through EBSCO Host).
4. Ideas Repec (through EBSCO Discovery).
5. PAIS Index (through ProQuest).
6. ProQuest Dissertations & Theses Global.

To ensure maximum coverage of gray literature, we will also look for studies in the web sites of the following organizations.

1. 3ie database of impact evaluations.
2. African Development Bank.
3. Agricultural Technology Adoption Initiative (ATAI).
4. American Economic Association RCT Registry.
5. Asian Development Bank.
6. Inter‐American Development Bank.
7. Organizations for the Economic Co‐operation and Development (OECD).
8. UK Department for International Development (DFID).
9. US Agency for International Development (USAID).
10. World Bank Group.

In addition, we will also search for relevant papers in the following specialized journals:

1. Economics of Innovation and New Technology.
2. Industrial and Corporate Change.
3. Journal of Development Effectiveness.
4. Research Policy.
5. The Journal of Technology Transfer.

Our search will use Boolean operators to express the following requirements:

1. The papers should include terms related to the outcomes in the title or abstract.
2. The papers should include terms related to the methodology in the title, the abstract or the main text.
3. The papers should be dated in the year 2000 or later. As do Piza et al. (2016), we impose this limit because we will focus on studies using impact evaluation techniques, which have been widely adopted in development economics since that time. Even though it is possible that some papers on technology adoption were produced before this year, we expect that these will not address endogeneity issues in the way required to be included in this review. Therefore, allowing papers before 2000 in the search would increase the number of hits and the time required to sift through them, without adding more than a few papers, if any.

Note that in order to keep the electronic search as broad as possible, the electronic search will not differentiate between technological or economic outcomes. Following EBSCO host's search syntax, the Boolean expression to implement the search will be the following (in addition, a date filter for documents after 2000 and language filter for English will be added):

(TI(*outcomes*) OR AB(*outcomes*)) AND (TI(methods) OR AB(methods)OR TX(methods))

where:

*outcomes* = ((technolog* OR manag* OR innovat* OR practice*) W4 (adopt* OR diffus* OR chang* OR alter*)) OR productivity OR “output per” OR (unit* W4 cost)

*methods* = (“quasi experiment*” or quasi‐experiment* or “random* control* trial*” or “random* trial*” or RCT or (random* N3 allocat*) or match* or “propensity score” or PSM or “regression discontinuity” or “discontinuous design” or RDD or “difference in difference*” or difference‐in‐difference* or “diff in diff” or “case control” or cohort or “propensity weighted” or propensity‐weighted or “interrupted time series” or “Control* evaluation” or “Control treatment” or “instrumental variable*” or heckman or IV or ((quantitative or “comparison group*” or counterfactual or “counter factual” or counter‐factual or experiment*) N3 (design or study or analysis)))

‘W4’ is EBSCO specific search syntax that stands for “within 4 words of each other”. Similarly, ‘N3’ stands for “near 3 words of each other”.

The exact syntax to be used in Econlit is presented in Appendix 1. It will be adapted to the different database providers as appropriate.

#### Manual search

In addition to the electronic search, we will conduct a manual search that entails three steps: First, we will search the references in the selected papers for additional references. Second, we will seek recommendations of additional references from experts and practitioners. Third, we will conduct google citation search of included studies.

### Description of methods used in primary research

In this section, we provide examples of papers that satisfy and do not satisfy the inclusion criteria of our review.

Atkin et al. (2017) studied the process of technology adoption and the role of the alignment of incentives between employers and employees. They carried out two randomized experiments with soccer ball producers in Pakistan. In the first one, they offered a new production technology to a randomly selected group of firms and found that adoption was very low: only five out of 35 technology recipients adopted after more than a year. The authors conjectured that the low level of adoption could be explained by the fact that, even though the new technology was better than the old one, the benefits of adoption were unevenly distributed between employers and employees. Adoption represented a cost for workers as they were paid by pieces processed and the new technology slowed them down during the adaptation period (the advantage of the new technology relied on reducing waste, but not in increasing the production speed). To test this hypothesis, the authors ran a second experiment. They randomly offered to pay bonuses to workers if the workers could demonstrate competence with the new technology. This treatment changed workers’ incentives (aligning them with the employers) and had a positive effect on the adoption probability. This study qualifies to be included in the review because i) the participants of the intervention are firms; ii) the interventions attempt to induce technology adoption in firms (that is explicitly stated in the paper); iii) technology adoption is one of the outcome variables the authors pay attention to; iv) in the experiments there is a set of firms that do not receive treatment; and v) the method used is a randomized control trial.

Beaman et al. (2014) study the adoption of better small change (bills and coins of low denomination that allow businesses to give change when making a transaction) management practices among firms in Kenya. To do so, the authors run two experiments. In the first one, the treatment was visiting the firms once per week and increasing their awareness about the importance of change management through a survey that highlighted the profit losses due to changeouts (i.e. not having sufficient change). In the second one, the treatment was providing information about amount of money lost by each firm due to this problem. In the first experiment, all the firms were treated at some point, but the timing of that treatment was randomly allocated, providing treatment and control groups in a given (intermediate) point in time. In the second experiment, the treatment allocation was random. They conclude that both interventions led to adoption of better change management practices and to a reduction in the sales lost due to changeouts. These study would be included in our review because i) the participants are firms; ii) the intervention attempts to change management practices, that are a form of technology; iii) technology adoption is one of their outcomes (they infer adoption by counting the number of changeouts they experience due only to the fact that they did not have bills of the right denomination); iv) the authors present effect estimates comparing treated firms with firms that have not received the interventions; and v) the method used is a randomized control trial.

Menzel (2016) studies the effect of an information intervention in Bangladeshi garment factories. The intervention was randomized at the level of production line and consisted of instructing a line chief with experience producing some garment to brief an inexperienced line chief about potential production problems. The author concludes that treated production lines saw an increase in productivity. This study would be included in our review given that i) the participants of the intervention are productive units; ii) the content of the intervention is the implementation of a new management practice (briefing between production lines); iii) even though the main results of the paper are intention‐to‐treat estimates of the effect of the intervention on productivity, the author recorded the actual treatment status (as evidenced in table 7); iv) the author presents estimates comparing treated and non‐treated production lines; and v) the method used is a randomized controlled trial

Castillo et al. (2014) study the impacts of the Argentinean FONTAR program, which offers subsidies for innovation activities, including technological upgrading. The authors estimate the effects of this intervention on employment, wages and exports using propensity score matching and difference‐in‐differences (dif‐in‐dif). This study fulfils the inclusion conditions regarding participants, methods, intervention and comparison; but fails the outcomes condition. In particular, the study does not report results regarding technology adoption. Instead, it just shows the link between the intervention and the final outcomes, omitting the intermediate ones. Consequently, this study would not be included in our review.

### Data extraction and analysis

#### Selection of studies

As indicated in section 3.2, the output of the electronic search process will be a broad set of *candidate papers*, including many articles that are not relevant for our review. Naturally, not all candidate papers will meet the criteria to be included in the review.

**Figure 2:**
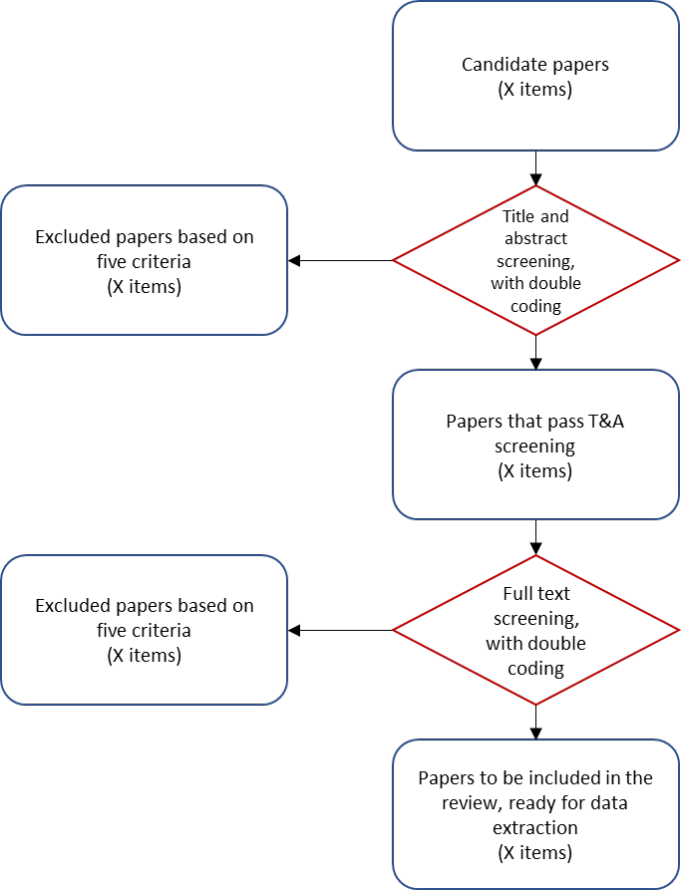
Screening procedure

We will use a two‐step screening procedure, covering all candidate papers after duplicates removed, to identify those that meet the criteria to be included in the review, see figure 2. First, all candidate papers will be screened based on title and abstract. Second, the papers that pass the title and abstract screening will be screened again based on the full article text. If a paper passes both screening stages, it will reach the data extraction phase and be included in the review. Note that the two‐step screening procedure will not exclude articles based on whether the reported estimates are usable in the review or not.

In each of the screening stages, the reviewer will have to determine whether a paper must be excluded or not based on the following criteria:

1. Exclude based on participants. The study should be excluded if the unit of analysis is not a productive unit. This means that the reported effects must be at the level of that unit.
2. Exclude based on intervention. The study should be excluded if it does not mention explicitly that the intervention has the potential of inducing technology adoption by a firm (as defined in subsection 3.1.2).
3. Exclude based on outcomes. The study should be excluded if it does not report the effect of the intervention on technology adoption (as defined in subsection 3.1.3). Note that at this point of the screening process we will not impose restrictions regarding the final outcomes.
4. Exclude based on study design. The study should be excluded if it does not use one of the methods described in subsection 3.1.5 (randomized control trial, instrumental variables, regression discontinuity, difference‐in‐difference, propensity score matching or synthetic control).
5. Exclude based on date. The study should be excluded if it is dated before the year 2000.

For the title and abstract screening, we will use a ‘double coding procedure’ for the first 700 papers coded by each reviewer, grouped in two batches of 350. The overall objective of double coding is to promote learning and increase consistency across reviewers. After reaching the 700 papers with double coding, each reviewer will continue the title and abstract screening with single coding. This means that each subsequent paper will be coded only once, by the responsible reviewer.

More specifically, the double coding procedure for title and abstract screening will be implemented as follows:

1. Using the program ‘EPPI reviewer 4’, a codeset called ‘Screen on title and abstract’ will be created, incorporating the five selection criteria indicated above and the option to‘include based on title and abstract’.
2. All candidate papers will be grouped in batches of 350 papers. The ‘allocation group I’ will be assigned to reviewers A and B.
3. Reviewers A and B will independently screen the papers in ‘allocation group I’ by reading their title and abstract. For each paper, each reviewer will mark if it should be excluded and select the criteria that applies. When appropriate, they will leave a note clarifying their decision (using button “info” right next to the exclusion flag).
4. After both reviewers have finished the screening of the ‘allocation group I’, their coding will be compared (using the “create comparison” button). The codes in agreement will be accepted (marked as “completed”). The reviewers will note the number of disagreements and solve them in a call. In the discussion, all doubts or errors will be identified and addressed such that each reviewer is learning and improving their screening process. The agreed codes will be accepted.
5. Subsequently, the next batch of 350 papers, ‘allocation group II’, will be assigned for title and abstract screening to reviewers A and B, and the procedures described in points 3 and 4 above will be repeated.
6. Subsequently, ‘allocation group III’ will be assigned to reviewer A and ‘allocation group IV’ to reviewer B. Each reviewer will screen their respective new batch with single coding.

The sub‐set of papers that are not excluded in the title and abstract screening, will advance to the full text screening. The full text screening will be implemented using a new codeset in EPPI‐reviewer called ‘screen on full text’, which will include the same five exclusion criteria or flags. This time, to ensure consistency and learning across reviewers, we will implement the double coding procedure for the first 20 papers coded by each reviewer, grouped in two batches of 10.

#### Data extraction

The research team will extract the relevant information from each of the selected papers. Risk of bias assessment and impact estimates will be collected in duplicate and discrepancies discussed and solved between reviewers. The other variables will be collected only once. The information will be entered into an Excel spreadsheet for processing. The variables for data extraction are presented in Appendix 2.

#### Assessment of risk of bias

Once we have selected the studies for our review based on the criteria for inclusion and exclusion of studies (subsection 3.1), we will use a modified version of the 3ie risk of bias tool (version 2012) to assess the extend in which the studies address bias risks. The tool is a checklist to determine whether a study addresses bias threats in different dimensions. In our version of the tool we will consider five types of bias risks: selection bias and confounding, spillovers, selective outcome and analysis reporting, inference, and other sources of bias. The risk of bias analysis will be conducted per outcome.

For each of these dimensions, the question whether the study addresses the threat can receive one of three answers: “yes”, “unclear” or “no”. Similar to Piza et al. (2016) and Baird et al. (2013), we will classify the studies in three groups based on these answers:

- Low risk of bias: If “yes” for at least four dimensions.
- Medium risk of bias: If “yes” for at least three dimensions.
- High risk of bias: If “yes” in less than three dimensions,

To determine whether a study addresses each dimension of bias risk, we will consider a set of conditions. The answer will be based on whether those conditions are satisfied or not. The conditions and the scoring procedure are detailed in Appendix 3. The risk‐of‐bias assessment will be used to describe the quality of the evidence found.

The studies classification will be qualified with a prefix: “RCT” for randomized control trials; “QE” for other quasi‐experimental methods; and “NE” for non‐experimental methods. This prefix system will be useful to acknowledge the fact that, regardless of how well the analysis is implemented, different methods differ in their ability to control the risk of bias. For example, a well implemented randomized control trial has a stronger ability to control confounding factors than a well implemented study that uses propensity score matching. Hence, it would not be appropriate to put both in the same “Low” risk of bias category. Instead, it is more informative to indicate that the randomized control trial has a “RCT‐Low” risk of bias, and the matching analysis has a “NE‐Low” one.

#### Measures of treatment effect

Comparing different pieces of evidence about an intervention requires comparable effect size estimates. These comparable measures are usually not directly provided in the original study and need to be computed by the reviewer based in the information in the paper.

As indicated in Borenstein et al. (2009), when the outcomes are not directly measured in a common and meaningful scale, we will use the standardized mean difference (SMD). This measure re‐expresses the impact measure of a study relative to the outcome variability observed in that study. A positive value of the SMD indicates a positive impact of the intervention on the outcome. We will compute the SMD using the following formula:

$$SMD=\frac{TE}{S}$$

where TE is the treatment effect and ^*S*^ is the pooled standard deviations of the outcome in the population of firms that are part of the study.

To compute the SMD's standard error we will use the following expression:

$$SE(SMD)=\sqrt{\frac{N_{T}+N_{C}}{N_{T}N_{C}}+\frac{SMD^{2}}{2(N_{T}N_{C})}}$$

where ^*N*^_*T*_ and ^*N*^_*C*_ are the sample size of the treatment and control groups.

In cases in which the pooled standard deviation is not reported, we will compute the SMD using the t‐statistic (^*t*^) of the treatment effect estimate.

$$SMD=t\sqrt{\frac{N_{T}+N_{C}}{N_{T}N_{C}}}$$

If the sample size is not presented by group, we will estimate the SMD and its standard error with the following formulas.

$$SMD=\frac{2t}{\sqrt{N}}$$

$$SE\;(SMD)=\sqrt{\frac{4}{N}+\frac{SMD^{2}}{4N}}$$

where ^*N*^ is the total sample size of the study.

For the impact of dichotomous variables on dichotomous variables, whenever possible, we will also compute the risk ratio (RR). The RR is the ratio between the probability of the outcome variable taking value 1 under the treatment and the probability of it taking value 1 without the treatment. When the intervention increases the probability of the outcome variable taking value 1, the RR is higher than 1. When the intervention decreases that probability, the RR takes value between 0 and 1. Computing this quantity is straightforward when the results are reported in a two‐by‐two table. This is common practice some disciplines like the medical science, but it is not customary in social sciences. We expect to find the results mainly in the form of a regression coefficient. Then, we will compute RR using the following formula (Waddington et al. 2014):

$$RR=\frac{{\bar{Y}}_{C}+b}{{\bar{Y}}_{C}}$$

where ${\bar{Y}}_{C}$ is the average value of the outcome in the control group and ^*b*^ is the estimate of the regression coefficient of the treatment variable.

To compute the RR's standard error, we will use the following formula:

$$SE\;(RR)=\sqrt{S^{2}\left(\frac{1}{N_{T}{\left({\bar{Y}}_{C}+b\right)}^{2}}+\frac{1}{N_{C}{\bar{Y}}_{C}^{2}}\right)}$$

#### Methods for handling dependent effect sizes

When summarizing the effect estimates, we will treat different estimates from the same outcome‐intervention as a single study. This entails the need of collapsing results from different papers about the same intervention‐outcome into one single estimate, and also the need to collapse different estimates for the same outcome‐intervention in the same paper into one single estimate. We will do this in the following way: In the case of estimates different subpopulations (for example, firms from different sectors) or belonging to different points in time, we will collapse those estimates to their sample‐weighted average. The variance of this collapsed estimate will be computed using the following formula:

$$VAR(\sum a_{i}\;y_{i})=\sum a_{i}^{2}\;v_{i}+\sum_{i\neq j}a_{i}\;a_{j}\;r_{ij}\;v_{i}\;v_{j}$$

where ^*y*^_*i*_ are the effect sizes on different subpopulations or points in time ^*i*^, ^*ν*^_*i*_ are their variances, ^*r*^_*ij*_ is their correlation coefficient and ^*a*^_*i*_ are weights in the sample‐weighted average. When summarizing estimates for different subpopulations, we will assume that the correlation between estimates is zero. When adding estimates for a given population for different points in time, we will assume correlation equal to one.

In the case of estimates coming from different specifications (for example, from regressions with different control variables), we will consider the effect estimate that is preferred by the authors.

#### Unit of analysis issues

As stated before, we will focus on papers that take the firm as the unit of analysis. However, the unit of analysis might not coincide with the level of experimental or quasi‐experimental variation. For instance, a study might report estimates of firm‐level impacts of a technology adoption program that was assigned to the firms in selected districts. In this case, the treatment assignment status varies at district level instead of firm‐level. Studies like this should consider the intracluster correlation when computing the standard errors of their effect estimates. Fortunately, this practice has been increasingly adopted in social sciences with the use of cluster‐robust standard errors. If we find a study that do not consider the intracluster correlation, we will contact the authors and will correct the reported standard errors using the following formula (Cochrane Handbook, section 16.3.4):

$$SE\prime =SE\sqrt{1+(m+1)ICC}$$

where ^*SE*^ and ^*SE′*^ are corrected and original (uncorrected) standard errors ^*m*^, is the average number of observations per cluster and is the intracluster correlation.

#### Dealing with missing data

When relevant information is not reported (like the pooled standard deviations or the intracluster correlation), we will contact the authors of the studies to ask for it.

#### Assessment of heterogeneity

We will use the I‐squared statistic to estimate relative heterogeneity (the *proportion* of the observed variance ‐across effect estimates‐ that is due to differences in the true effect size) and the tau‐squared statistic to estimate the absolute heterogeneity (the variance of the true effects).

### 3.5 Data synthesis

#### Quantitative data

To summarize the evidence from the included papers, meta‐analysis will be used. In particular, we will use a random effects model to compute the summary effect of the interventions on the intermediate outcome. The summary effect and effect sizes of the individual studies will be presented in forest plots. Because we are only including papers that report effects about technology adoption, it is not possible to build a summary measure for the impact on the final outcomes (the sample of studies would not be representative). The effect size for these outcomes will be presented using a forest plot, but without including a summary measure.

To explore the heterogeneity of the effect of the interventions on technology adoption (review questions 2), and of technology adoption on the final outcomes (review question 3), we will use meta‐regression (moderator analysis).

#### Qualitative data

This review will focus in quantitative data. Qualitative information embedded in the quantitative studies will be used to understand the implementation of the interventions, which will inform the answers to the questions in the modified 3ie risk of bias tool.

### Review authors, support, and timeframe

#### Names and affiliations

**Lead review author:**

**Table:** 

**Name:**	**Eric Verhoogen**
Title:	Professor
Affiliation:	Economics and SIPA, Columbia University
Address:	20 W. 118th St., Room 1022, MC 3308,
City, State, Province or County:	New York, NY
Postal Code:	10027
Country:	USA
Email:	eric.verhoogen@columbia.edu
	
**Co‐authors:**	
**Name:**	**David Alfaro‐Serrano**
Title:	PhD Candidate
Affiliation:	Columbia University
Address:	420 W. 118^th^ St., MC 3308
City, State, Province or County:	New York, NY
Postal Code:	10027
Country:	USA
Email:	da2628@columbia.edu
	
**Name:**	**Tanay Balantrapu**
Title:	Research Analyst
Affiliation:	World Bank Group
Address:	1818 H Street NW
City, State, Province or County:	Washington DC
Postal Code:	20433
Country:	USA
Email:	tbalantrapu@ifc.org
	
**Name:**	**Ana Goicoechea**
Title:	Senior Economist
Affiliation:	World Bank Group
Address:	1818 H Street NW
City, State, Province or County:	Washington DC
Postal Code:	20433
Country:	USA
Email:	agoicoechea@worldbank.org
**Team advisor:**	
**Name:**	**Xavier Cirera**
Title:	Senior Economist
Affiliation:	World Bank Group
Address:	1818 H Street NW
City, State, Province or County:	Washington DC
Postal Code:	20433
Country:	USA
Email:	xcirera@worldbank.org

#### Roles and responsibilities

Please give a brief description of content and methodological expertise within the review team. It is recommended to have at least one person on the review team who has content expertise, at least one person who has methodological expertise and at least one person who has statistical expertise. It is also recommended to have one person with information retrieval expertise. Please note that this is the *recommended optimal* review team composition.

- Content: All co‐authors
- Systematic review methods: Ana, David, Tanay, Xavi
- Statistical analysis: Tanay
- Information retrieval: David, Tanay

#### Declarations of interest

There are no conflicts of interest identified at this time.

#### Sources of support

The study will be funded by the Competitiveness Policy Evaluation Lab (ComPEL) of the World Bank Group.

#### Preliminary timeframe

We plan to submit a draft review by 30 October 2018 and the final draft review by 30 January2019.

#### Plans for updating the review

Any co‐author may consider updating the review in two years if the literature advances considerably.

### Author declaration

#### Authors’ responsibilities

By completing this form, you accept responsibility for preparing, maintaining and updating the review in accordance with Campbell Collaboration policy. The Campbell Collaboration will provide as much support as possible to assist with the preparation of the review.

A draft review must be submitted to the relevant Coordinating Group within two years of protocol publication. If drafts are not submitted before the agreed deadlines, or if we are unable to contact you for an extended period, the relevant Coordinating Group has the right to de‐register the title or transfer the title to alternative authors. The Coordinating Group also has the right to de‐register or transfer the title if it does not meet the standards of the Coordinating Group and/or the Campbell Collaboration.

You accept responsibility for maintaining the review in light of new evidence, comments and criticisms, and other developments, and updating the review at least once every five years, or, if requested, transferring responsibility for maintaining the review to others as agreed with the Coordinating Group.

#### Publication in the Campbell Library

The support of the Coordinating Group in preparing your review is conditional upon your agreement to publish the protocol, finished review, and subsequent updates in the Campbell Library. The Campbell Collaboration places no restrictions on publication of the findings of a Campbell systematic review in a more abbreviated form as a journal article either before or after the publication of the monograph version in Campbell Systematic Reviews. Some journals, however, have restrictions that preclude publication of findings that have been, or will be, reported elsewhere and authors considering publication in such a journal should be aware of possible conflict with publication of the monograph version in Campbell Systematic Reviews. Publication in a journal after publication or in press status in Campbell Systematic Reviews should acknowledge the Campbell version and include a citation to it. Note that systematic reviews published in Campbell Systematic Reviews and co‐registered with the Cochrane Collaboration may have additional requirements or restrictions for co‐publication. Review authors accept responsibility for meeting any co‐publication requirements.

**I understand the commitment required to undertake a Campbell review, and agree to publish in the Campbell Library. Signed on behalf of the authors:**

**Form completed by: Eric, David, Tanay and Ana**

**Date: July 19, 2018**

## Footnotes

1 The literature on bias technical change have highlighted that, even though the level of wages might increase after the adoption of better technologies, the relative price of different inputs (labor versus capital, or unskilled labor versus skilled labor) might move in the opposite direction.

2 In other words, the intermediate outcomes in the theory of change are the primary outcomes of the review and the final outcomes of the theory of change are the secondary outcomes of the review. To prevent confusion, this terminology will be avoided throughout the review. The expressions “intermediate outcome” and “final outcome” will be preferred.

## ECONLIT SEARCH SYNTAX

The following search syntax was used in the Econlit database:

(TI(((technolog* OR manag* OR innovat* OR practice*) W4 (adopt* OR diffus* OR chang* OR alter*)) OR productivity OR “output per” OR (unit* W4 cost)) OR AB(((technolog* OR manag* OR innovat* OR practice*) W4 (adopt* OR diffus* OR chang* OR alter*)) OR productivity OR “output per” OR (unit* W4 cost))) AND (TI((“quasi experiment*” or quasi‐experiment* or “random* control* trial*” or “random* trial*” or RCT or (random* N3 allocat*) or match* or “propensity score” or PSM or “regression discontinuity” or “discontinuous design” or RDD or “difference in difference*” or difference‐in‐difference* or “diff in diff” or “case control” or cohort or “propensity weighted” or propensity‐weighted or “interrupted time series” or “Control* evaluation” or “Control treatment” or “instrumental variable*” or heckman or IV or ((quantitative or “comparison group*” or counterfactual or “counter factual” or counter‐factual or experiment*) N3 (design or study or analysis)))) OR AB((“quasi experiment*” or quasi‐experiment* or “random* control* trial*” or “random* trial*” or RCT or (random* N3 allocat*) or match* or “propensity score” or PSM or “regression discontinuity” or “discontinuous design” or RDD or “difference in difference*” or difference‐in‐difference* or “diff in diff” or “case control” or cohort or “propensity weighted” or propensity‐weighted or “interrupted time series” or “Control* evaluation” or “Control treatment” or “instrumental variable*” or heckman or IV or ((quantitative or “comparison group*” or counterfactual or “counter factual” or counter‐factual or experiment*) N3 (design or study or analysis))))OR TX((“quasi experiment*” or quasi‐experiment* or “random* control* trial*” or “random* trial*” or RCT or (random* N3 allocat*) or match* or “propensity score” or PSM or “regression discontinuity” or “discontinuous design” or RDD or “difference in difference*” or difference‐in‐difference* or “diff in diff” or “case control” or cohort or “propensity weighted” or propensity‐weighted or “interrupted time series” or “Control* evaluation” or “Control treatment” or “instrumental variable*” or heckman or IV or ((quantitative or “comparison group*” or counterfactual or “counter factual” or counter‐factual or experiment*) N3 (design or study or analysis)))))

## ARTICLE CODING AND DATA EXTRACTION

**Table:**

**Publication details**	Author(s) Year of publication Full citation Publication type: journal article, working paper, book Author(s) affiliation(s) Funding sources for the research
**Context of intervention or program**	Region: EAP, ECA, LAC, MNA, SAR, SSA Country Declared goal of the intervention If it is a government deliberate intervention, ministry in charge If it is a deliberate intervention, agency in charge of implementation Where did the intervention take place? Period considered in the study Location: urban, rural Funding source(s) for the intervention Total cost of the intervention (US dollars)
**Population in the study**	If the intervention targets a specific type of unit, definition of target Unit of analysis in the study Average annual output of the productive units Average annual sales of the productive units Average employment of the productive units Sector(s) of operation Fraction of the productive units owned, led, or operated by female individuals. Average fraction of high‐skill workers in units of production Average fraction of female workers in units of production Ownership composition of productive units
**Intervention details**	Name of the intervention (if any) and brief description Does it include an indirect financial support component? Does it include a direct financial support component? Does it include other direct support component? Does it include a component affecting regulation and standards?
**Study design**	Methodology: Description of sample selection procedure, including units and sample size for treatment, exposed, and comparison groups If reported separately, treatment group sample size If reported separately, comparison group sample size If reported separately, indirectly exposed group sample size Total sample size If reported separately, attrition in treatment group If reported separately, attrition in comparison group If reported separately, attrition in indirectly treated group Overall attrition Frequency of outcome data collection Total number of data collection periods including baseline Baseline period Intervention periods Follow up periods Duration of intervention Spillovers Contamination
Data for analysis (per treatment‐outcome pair)	Treatment variable Treatment variable's unit of measurement Outcome variable Outcome variable's unit of measurement Technology adoption outcome? (yes/no) Observations Sample size (N) If reported separately, sample size of treatment group If reported separately, sample size of comparison group If reported separately, sample size of indirectly exposed group MeanC_Pre: Pre‐intervention comparison group mean MeanC_Post: Post‐intervention comparison group mean sdC_Pre: Pre‐intervention comparison group standard deviation sdC_Post: Post‐intervention comparison group standard deviation MeanT_Pre: Pre‐intervention treatment group mean MeanT_Post: Post‐intervention treatment group mean sdT_Pre: Pre‐intervention treatment group standard deviation sdT_Post: Post‐intervention treatment group standard deviation If outcome and treatment are dichotomous, success in treatment group If outcome and treatment are dichotomous, success in comparison group If outcome and treatment are dichotomous, failure in treatment group If outcome and treatment are dichotomous, failure in comparison group sdpooled_pre: pooled standard deviation Type of treatment effect estimated: ITT, ATET, ATE, LATE Effect estimate Effect estimate SE t‐statistic If significance is presented in the paper, level of significance of the test presented (usually reported with stars). If p‐value of significance test is presented in paper, p‐value Estimation model Effect size adjustment Total number of regressors used (including treatment variables) R‐square Chi‐square F‐test for the test of joint significance of all regressors Is the impact restricted to a sub‐group of firms SMD SE(SMD) t If outcome and treatment are dichotomous, RR If outcome and treatment are dichotomous, SE(RR) Risk of bias: selection bias and confounding (yes, no, unclear) Risk of bias: spillovers (yes, no, unclear) Risk of bias: selective outcome and analysis reporting (yes, no, unclear) Risk of bias: Inference (yes, no, unclear) Risk of bias: Other sources of bias (yes, no, unclear) Overall risk of bias assessment

## RISK OF BIAS ASSESSMENT TOOL

***1. To assess risk of selection bias and confounding the conditions depend on the method used by the study*.**

*If the study uses randomized control trial:*

Condition 1: The study explicitly indicates that the assignment was done at random, or the study describes a random component as part of the assignment procedure.

Condition 2: If the assignment is done at group level, all the groups were assigned at the start of the study.

Condition 3: The study reports baseline characteristics of the treatment and control groups, and they are overall statistically similar.

Condition 4: If there is some difference in baseline characteristics between treated and control groups, this difference is controlled for when estimating the treatment effects.

Condition 5: If there is attrition, the study shows that the loss of sample units can be considered random.

Score YES if conditions 1 through 5 hold.

Score UNCLEAR if conditions 1 and 3 hold, and the remaining conditions either hold or there is not enough information to determine that.

Score NO otherwise.

If the study uses a regression discontinuity design:

Condition 1: The study indicates that the allocation is based on a threshold in a continuous variable.

Condition 2: The study argues convincingly that the participants cannot manipulate the assignment variable.

Condition 3: The estimation is made using non‐parametric techniques and observations weights are chosen using a standard method for regression discontinuity (for example, kernels with IK bandwidth or CCT bandwidth).

Condition 4: The study shows that baseline characteristics are overall continuous around the assignment threshold.

Condition 5: If some baseline characteristic shows a discontinuity around the assignment threshold, this difference is controlled for when estimating the treatment effects.

Score YES if conditions 1 through 5 hold.

Score UNCLEAR if conditions 1 and 2 hold; and condition 3 does not hold, but the following conditions does: “the estimation is made using parametric techniques and robustness to the degree of the polynomial used is shown”; and the remaining conditions either hold or there is not enough information to determine that.

Score NO otherwise.

If the study uses instrumental variables:

Condition 1: The study argues convincingly that the instruments affect the outcome variable only through the endogenous regressor (exclusion restriction).

Condition 2: The study shows the first stage regression and its F‐statistic is at least 10.

Score YES if conditions 1 and 2 hold.

Score UNCLEAR if condition 1 holds and condition 2 does not, but instead the following condition holds: “the study shows the first stage regression and each instrument is statistically significant”.

Score NO otherwise.

If the study uses difference‐in‐difference:

Condition 1: The study argues convincingly that the timing of the treatment does not correlate with characteristics of the treatment group.

Condition 2: The study shows that the outcome variables’ trends are parallel before the introduction of the treatment.

Condition 3: If there is attrition, the study shows that the loss of sample units can be considered random.

Condition 4: Differences in covariates between the treatment and control groups, are controlled for when estimating the treatment effects.

Score YES if conditions 1 through 4 hold.

Score UNCLEAR if conditions 1 and 2 hold, and the remaining conditions either hold or there is no enough information to determine that.

Score NO otherwise.

If the study uses a statistical control technique (for example, propensity score matching):

Condition 1: The study describes the control method used and it is based on baseline characteristics that are relevant to explain participation.

Condition 2: The study shows that baseline characteristics that are not used for control are overall statistically similar different treatment groups.

Condition 3: If the study uses propensity score matching, only observations in the common support are used for estimation.

Score YES if conditions 1 and 3 hold.

Score UNCLEAR if condition 1 holds, and conditions 2 and 3 either hold or there is no enough information to determine that.

Score NO otherwise.

If the study uses synthetic control:

Condition 1: The study shows that the synthetic control matches the characteristics of the treated units before the treatment.

Condition 2: The study shows robustness to the weights used to measure the difference between the characteristics of the treated unit and the synthetic control.

Score YES if conditions 1 and 2 hold.

Score UNCLEAR is condition 1 holds.

Score NO otherwise.

***2. To assess whether bias risk derived from potential spillovers is properly addressed or not, we will consider the following condition:***

Condition 1: It is unlikely that the intervention spills over control units (including general equilibrium effects).

Score YES if condition 1 holds.

Score UNCLEAR if there is no enough information to determine whether condition 1 holds or not.

Score NO if condition 1 does not hold.

***3. To assess if the risk of selective outcome and analysis reporting is properly addressed, we will consider the following conditions:***

Condition 1: There is no evidence that outcomes were selectively reported (for example, all the relevant outcomes included in the data sources described are included in the results section).

Condition 2: The study presents specification tests (for example, multicolinearity tests or robustness of the results to the inclusion of different sets of covariates).

Score YES if conditions 1 and 2 hold.

Score UNCLEAER if there is no enough information to determine whether conditions 1 and 2 hold.

Score NO otherwise.

***4. To assess if risks related to inference are properly addressed, we will consider the following conditions:***

Condition 1: Heteroskedasticity‐robust estimators of the variance are used.

Condition 2: If it is reasonable to think that there might be lack of independence between observations, this is taken into account when computing the estimate's standard errors.

Condition 3: If the assignment of treatment was done at group level, cluster‐robust estimators of variance are used.

Condition 4: If the study does not find statistically significant effects despite large point estimates, the authors argue convincingly that the statistical power of the significance test is adequate.

Score YES if conditions 1 through 4 hold.

Score UNCLEAR if condition 1 holds, but there is not enough information to determine whether conditions 2 to 4 hold.

Score NO otherwise.

***5. To assess if other sources of bias are properly handled, we will use the following conditions:***

Condition 1: There are no concerns about placebo effects or courtesy bias on self‐reported outcomes.

Condition 2: If the data is collected in the context of this specific study, the study argues convincingly that the monitoring process did not affect behavior (Hawthorne effect).

Condition 3: Baseline information has not been collected retrospectively.

Score YES if conditions 1 through 3 hold.

Score UNCLEAR if there is no enough information to determine whether conditions 1 through 3 hold.

Score NO otherwise.
